# Preanalytical protocol for fresh solid tumor biospecimens

**DOI:** 10.21203/rs.3.rs-3911742/v1

**Published:** 2024-02-01

**Authors:** Areesha A. Charania, Aman G. Pokal, Dana R. Zuaiter, Mozaffarul Islam, Alex Kim, Karim I. Budhwani

## Abstract

Nearly seventy percent of diagnostic lab test errors occur due to variability in preanalytical factors. ^1–4^ These are the parameters involved with all aspects of tissue processing, starting from the time tissue is collected from the patient in the operating room, until it is received and tested in the laboratory. While there are several protocols for transporting fixed tissue, organs, and liquid biopsies, such protocols are lacking for transport and handling of live solid tumor tissue specimens. There is a critical need to establish preanalytical protocols to reduce variability in biospecimen integrity and improve diagnostics for personalized medicine. ^2,5^ Here, we provide a comprehensive protocol for the standard collection, handling, packaging, cold-chain logistics, and receipt of solid tumor tissue biospecimens to preserve tissue viability.

### BEFORE YOU BEGIN

*Permissions*

Patient permission for the tumor tissue study should be obtained. Tumor tissue specimens were collected from patients who underwent operative surgery at the Ohio State University James Comprehensive Cancer Center (OSUCCC), located in Columbus, Ohio. All patients provided informed consent for the collection and donation of the specimens.

*Notes*

In order to distinguish laboratory at the collection site from the laboratory at the analysis site, in this protocol, the laboratory at the collection site will be referred to as OSUCCC-Lab and the laboratory at the analysis site will be referred to as CerFlux-Lab. Note that this convention is adopted for reader convenience and clarity only. The protocol is designed for use between any two sites.

### KEY RESOURCES TABLE

| Reagent or Resource | Source | Identifier |
|---|---|---|
| *Chemicals, peptides, and recombinant proteins* | | |
| $ddH_2O$ | | |
| DMEM/F-12 50/50, with L-glutamine and without phenol red (1X) | Corning | REF: 16-405-CV |
| DMEM/F-12 50/50, with L-glutamine and phenol red (1X) | Corning | REF: 10-090-CV |
| Fetal Bovine Serum (FBS) | Corning | REF: 35-015-CF |
| GlutaMAX (100X) | Gibco | REF: 35050-061 |
| Paraformaldehyde, 4% | Thermo Scientific | CAT#: J61899 |
| Penicillin-Streptomycin (100X) | Corning | REF: 30-002-CI |
| Phosphate-Buffered Saline, USP sterile (10X) | VWR | CAT#: 97063-660 |
| Polyamine supplement (1000X) | Sigma-Aldrich | SKU: P8483 |

| | | |
|---|---|---|
| Primocin | InvivoGen | CAT#: ant-pm-1 |
| Reagent alcohol, v/v, 70% | RICCA | CAT#: 2546.70-5 |
| Trace element A | Corning | REF: 25-021-CI |
| Trace element B | Corning | REF: 25-022-CI |
| *Software and algorithms* | | |
| Govee Home application | Shenzhen Intellirocks Tech Co. Ltd. | Version: 6.0.02 |
| Microsoft Office 365 | Microsoft Corporation | |
| *Other* | | |
| Analytical balance | U.S. Solid | |
| Biohazard specimen bags | VWR | CAT#: 11215-684 |
| Biosafety cabinet | Baker Co. | |
| Bottle-top vacuum filter (500 mL) | VWR | CAT#: 10040-436 |
| Centrifuge tubes (1.5 mL) | VWR | CAT#: 16466-046 |
| ClipTip tips (1000 uL) | Thermo Scientific | CAT#: 14-488-010 |
| Conical tubes (50 mL) | VWR | CAT#: 21008-178 |
| Conical tubes (5 mL) | Globe Scientific | CAT#: 111580BS |
| Electronic single channel pipette (15-1250 uL) | Thermo Scientific | CAT#: 14-3879-53BT |
| Foam container | Polar Tech | CAT#: 204/T12C |
| Forceps | | |
| Freezer (-20°C) | Whirlpool | |
| Fridge (4°C) | Whirlpool | |
| Glass bead sterilizer | VWR | CAT#: 75999-324 |
| Glass Petri dish | VWR | CAT#: 470313-346 |
| Ice packs | Nordic Ice | CAT#: NB15 |
| Kimwipes | Kimberly-Clark | |
| Label maker | Brother | CAT#: PT-D210 |
| Packaging tape | UPS | |
| Parafilm “M” | Sigma-Aldrich | SKU: P7668 |
| Pipette aid | Drummond | |
| Plastic Petri dish | VWR | |
| Ruler scale | | |
| Serological pipette (10 mL) | VWR | CAT#: 75816-100 |
| Serological pipette (50 mL) | VWR | CAT#: 75816-088 |
| Scalpel | | |
| Scalpel blade (No. 10) | VWR | CAT#: 76457-442 |
| Transportation boxes | Polar Tech | CAT#: 204/T12C |
| UV sterilizer | | |
| Vacuum filter (150 mL) | VWR | CAT#: 10040-460 |
| Wi-Fi Thermo-Hygrometer | Govee | REF: 210-166043 |

## MATERIALS AND EQUIPMENT

*Transport media*

| Reagent | Amount |
|---|---|
| DMEM/F-12 with L-glutamine and phenol red | 500 mL |
| FBS | 25 mL |
| GlutaMAX 100X | 5 mL |
| Pen-Strep | 5 mL |
| Polyamine supplement 1000X | 125 mL |
| Primocin | 1 mL |
| Trace element A | 125 mL |
| Trace element B | 250 mL |
| **Total** | **1036 mL** |

Sterilize the solution using a 500 mL capacity vacuum filter. Store 50 mL aliquots at 4°C; stable for up to 12 months; reusable.

*Buffer solution*

| Reagent | Amount |
|---|---|
| $ddH_2O$ | 45 mL |
| PBS (10X) | 5 mL |
| **Total** | **50 mL** |

Sterilize the solution using a 150 mL capacity vacuum filter into a 50 mL conical tube. Store at 4°C; stable for up to 12 months; reusable.

*Washing media*

| Reagent | Amount |
|---|---|
| DMEM/F-12 with L-glutamine, without phenol red | 500 mL |
| FBS | 50 mL |
| GlutaMAX 100X | 5 mL |
| Pen-Strep | 5 mL |
| Primocin | 1 mL |
| **Total** | **561 mL** |

Sterilize the solution using a 500 mL capacity vacuum filter. Store 50 mL aliquots at 4°C; stable for up to 12 months; reusable.

*Tissue procurement kit*

| Reagent or resource | Amount |
|---|---|
| 1.5 mL centrifuge tube with 4% PFA | 1 mL |
| 5 mL conical tube with transport media | 3.5 mL |

| | |
|---|---|
| Biohazard specimen bag | 2 |
| Foam container | 1 |
| Govee Wi-Fi Thermo-Hygrometer | 1 |
| Reusable ice pack | 3 |
| Tissue specimen collection form | 1 |
| Transport box | 1 |

## STEP-BY-STEP METHOD DETAILS

### *Packaging and shipping tissue procurement kits to collection facility*

This first segment of the protocol describes the preanalytical parameters involved in sending tissue procurement kits to the collection facility. **Note**: To ensure consistency in packaging materials and procedures, fully assembled tissue procurement kits containing all materials needed for biospecimen storage and transportation are shipped from the CerFlux-Lab to the tissue collection facility.

**Caution**: Strict aseptic techniques must be implemented when preparing and handling media and other reagents. Preparing and packaging tissue procurement kits for shipment to collection facility must be done in a sterile environment.

1. Prepare transport media in a sterile biosafety cabinet. **Note**: At a minimum, biosafety hood must be sterilized by exposure to UV for about 15 minutes followed by wiping down with 70% ethanol.

**Caution**: Ensure biologic and reagent substances are not present in the biosafety cabinet during sterilization.

a. Prepare transport media as detailed in **Materials and equipment**.

b. Use a pipette aid and sterile 10 mL serological pipette to transfer 3.5 mL into a sterile, labeled 5 mL conical tube.

c. Aliquot remaining transport media into sterile 50 mL conical tubes using a pipette aid and sterile 50 mL serological pipettes. Label and store in a sterile fridge at 4°C for future use.

2. Use 1250uL electronic single channel ClipTip pipette with sterile 1000uL ClipTip tips to dispense 1 mL of sterile 4% PFA into a sterile, labeled 1.5 mL centrifuge tube.

3. Spray exteriors of both the 5 mL conical tube, containing transport media, and the 1.5 mL centrifuge tube, containing PFA, with 70% ethanol; wipe down with Kimwipes;

4. Place both tubes in a sterile biohazard specimen bag and seal shut.

5. Prepare tissue procurement kit in a sterile environment.

a. Open the foam container and spray the interior with 70% ethanol.

b. Spray one reusable ice pack with 70% ethanol and place it on the bottom of the box.

c. Spray biohazard specimen bag containing both tubes and Govee Wi-Fi Thermo-Hygrometer with 70% ethanol; place them side-by-side onto ice pack.

d. Spray two reusable ice packs with 70% ethanol and insert them on either side of the biohazard bag and Govee; angle ice packs to cover the top.

e. Close the lid of the foam container and spray the exterior with 70% ethanol.

f. Seal foam container using clear packaging tape.

g. Spray interior of transport box with 70% ethanol and place foam container inside.

h. Insert the tissue specimen collection form inside a biohazard bag and place on top of the foam container.

i. Seal transport box using clear packaging tape and attach shipping label to the outside.

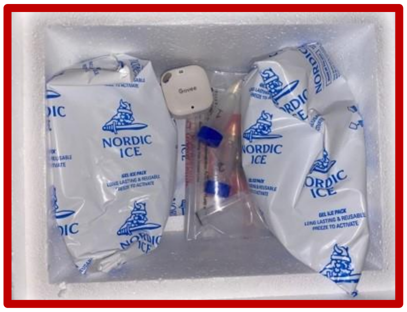

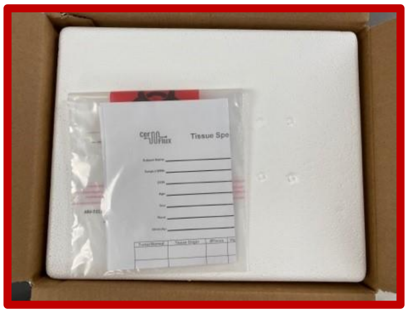

6. Notify the collection facility of package shipment.
7. Send return shipping label to collection facility electronically.

### *Receiving tissue procurement kit at collection facility*

This second step of the protocol focuses on the preanalytical parameters involved in receiving and storing the contents of the tissue procurement kits.

**Caution**: The handling of the tissue procurement kit must be done in a sterile environment.

1. Spray the exterior of the transport box with 70% ethanol and open it in a sterile environment.
2. Retrieve biohazard bag containing tissue specimen collection form and spray exterior with 70% ethanol before opening. **Note**: This form should be completed at the time of biospecimen collection.
3. Remove the foam container and spray its exterior with 70% ethanol before opening.
4. Remove the ice packs and sterilize with 70% ethanol before storing in freezer at –20°C for reuse.
5. Retrieve biohazard specimen bag containing media and PFA tubes; spray the exterior of the bag with 70% ethanol before unsealing.
6. Spray the exteriors of both tubes with 70% ethanol before examining for damage or contamination.
7. Store 5 mL conical tube containing transport media in a sterile environment at 4°C until use.
8. Store 1.5 mL centrifuge tube containing 4% PFA in a sterile environment at ambient room temperature until use.
9. Sterilize transport box and Govee Wi-Fi Thermo-Hygrometer with 70% ethanol and retain for shipping back to CerFlux-Lab.
10. Notify CerFlux-Lab of package receipt.
11. Ensure return shipping label has been received electronically.

### *Collecting and handling biospecimens at collection facility*

The third segment of this protocol describes the collection of tumor tissue from patients in the operating room.

**Caution**: Aseptic techniques must be implemented for all surgical procedures and handling of biospecimens in the operating room.

1. Under IRB approved protocol, consent and enroll patients with a diagnosis of peritoneal metastasis from colorectal cancer who are undergoing standard of care cytoreductive surgery.
2. Prep patients and their operative site under standard protocol.
3. Following entry into the abdominal cavity, individually resect peritoneal metastases and immediately place them into a biospecimen container.
4. Within 30 minutes of specimen resection, research personnel should transport the biospecimens (on ice) to OSUCCC-Lab.

### *Packaging and shipping of biospecimens from the collection facility*

The fourth step of the protocol focuses on the preanalytical parameters involved in shipping fresh solid tumor tissue from OSUCCC-Lab. **Note**: All specimens should be shipped to CerFlux-Lab within 12 hours of collection; all specimens must be received at CerFlux-Lab within 24 hours of shipment.

**Caution**: Aseptic techniques must be implemented when handling biospecimens. Preparing and packaging the tissue procurement kit for shipment must be done in a sterile environment.

1. In OSUCCC-Lab, biospecimens must undergo appropriate de-identification, labeling, and processing.
2. Place fresh solid tumor tissue biospecimens in a glass Petri dish and wash with 1X PBS.
3. Cut a 2 mm x 2 mm piece from a tumor specimen and immediately place into the 1.5 mL centrifuge tube containing 1 mL of 4% PFA. **Note**: This fixed specimen will be used for histological and correlative studies.
4. Immediately place all remaining tumor specimens into the 5 mL conical tube containing 3.5 mL of transport media.
5. Label both tubes with biospecimen identification details. **Note**: These labels should only identify the specimen and should not contain any patient identification information.
6. Seal both tubes with Parafilm and spray exterior of tubes with 70% ethanol and wipe down.
7. Place tubes into the provided biohazard specimen bag; spray exterior of bag with 70% ethanol and wipe down.
8. Prepare transportation box as detailed under *Packaging and shipping tissue procurement kits to collection facility,* steps 5a-i.
9. Send package to appropriate delivery provider for overnight shipping.
10. Notify CerFlux-Lab of shipment.

### *Receiving and handling biospecimens at analysis facility*

The fifth step of this protocol describes the preanalytical parameters involved with receiving, storing, and handling tumor tissue after it is received at CerFlux-Lab.

**Caution**: Aseptic techniques must be implemented when handling biospecimens in the laboratory. All procedures must be done in a sterile environment.

1. Spray the exterior of the transport box with 70% ethanol and open it in a sterile environment.
2. Retrieve biohazard bag containing completed tissue specimen collection form and spray exterior before opening. Confirm all information on the form and document into laboratory records.
3. Remove the foam container and spray the exterior with 70% ethanol before opening.
4. Remove the ice packs and sterilize with 70% ethanol before storing in freezer at –20°C for reuse.
5. Retrieve biohazard specimen bag and spray with 70% ethanol before unsealing.
6. Spray the exteriors of both tubes with 70% ethanol before examining for damage or contamination. Ensure tubes are properly labeled.
7. Confirm all items were maintained at the proper transport temperature and humidity by the Govee Wi-Fi Thermo-Hygrometer data.
8. Sterilize the thermo-hygrometer with 70% ethanol and retain for future use.
9. Handle fixed solid tissue biospecimens in a sterile biosafety cabinet. **Note**: At a minimum, biosafety hood must be sterilized by UV exposure for 15 minutes followed by wiping down with 70% ethanol.

**Caution**: Ensure biologic and reagent substances are not present in the biosafety cabinet during sterilization.

a. Allow tissue to be appropriately fixed by storing the 1.5 mL centrifuge tube, containing the fixed tissue specimen in 4% PFA, in a sterile environment at ambient room temperature for 24 hours.

b. Sterilize a glass Petri dish with 70% ethanol; once dry, sterilize in a UV hood for 10 minutes. Bring the dish into the biosafety cabinet.

c. Prepare 1X PBS solution as detailed in **Materials and equipment**.

d. Using forceps, remove the fixed biospecimen from the tube and place it into the glass Petri dish. **Note**: Forceps should be sterilized in a glass bead sterilizer at 260°C then acclimated to ambient temperatures before use.

e. Use a pipette aid and sterile 10 mL serological pipettes to wash biospecimen three times with 3 mL of ambient 1X PBS.

f. Place biospecimen into a labeled, sterile 1.5 mL centrifuge tube containing 1 mL of ambient 1X PBS.

g. Store tube in a sterile environment at ambient room temperature until histological and correlative analyses.

10. Handle fresh solid tissue biospecimens in a sterile biosafety cabinet. **Note**: At a minimum, biosafety hood must be sterilized by exposure to UV for about 15 minutes followed by wiping down with 70% ethanol.

**Caution**: Ensure biologic and reagent substances are not present in the biosafety cabinet during sterilization.

a. Sterilize a glass Petri dish with 70% ethanol; once dry, sterilize in a UV hood for 10 minutes. Bring the dish into the biosafety cabinet and keep cold using sterile ice packs.
   b. Prepare 1X PBS solution as detailed in **Materials and equipment**.
   c. Remove the biospecimen from the 5 mL tube using a pipette aid and sterile 10 mL serological pipette.
   d. Use a pipette aid and sterile 10 mL serological pipettes to thoroughly wash biospecimen five times with 5 mL of ice-cold 1X PBS to remove debris.
   e. Transfer specimen into a sterile, plastic, lidded Petri dish.
   f. Place Petri dish with biospecimen on a sterile analytical balance to accurately determine initial biospecimen weight. **Note**: Ensure balance has been tared to display 0.000 g using an empty Petri dish prior to weighing sample. Compare data provided in the tissue specimen collection form and document for laboratory records.
   g. Move Petri dish with biospecimen on a scale to image and ascertain proper dimensions. **Note**: Compare data provided in the tissue specimen collection form and document for laboratory records.
   h. Use forceps to transfer biospecimen back into the cold glass Petri dish. **Note**: Forceps should be sterilized in a glass bead sterilizer at 260°C then acclimated to ambient temperatures before use.
   i. Prepare washing media as detailed in **Materials and equipment**. Add 6 mL into the glass Petri dish.
   j. Hold biospecimen in place using forceps; section off a sample from the outside of the specimen using a scalpel and 10 blade. **Note**: Forceps, scalpel, and blade should be sterilized in a glass bead sterilizer at 260°C then acclimated to ambient temperatures before use.
      i. Place sectioned sample into a sterile, labeled 1.5 mL centrifuge tube containing 1 mL of 4% PFA. **Note**: This sample will be fixed for histological and correlative studies.
      ii. Follow steps 9a-g above for this specimen as well.

The remainder of the fresh solid tumor tissue biospecimen can be processed for analytical laboratory testing.

## VALIDATION

The temperature of the tissue procurement kit during transportation was monitored using Govee Wi-Fi Thermo-Hygrometer data; it maintained a consistent and stable temperature range of –5°C to 5°C throughout transport. Furthermore, to analyze the integrity of fresh solid tumor biospecimens, histological studies were conducted on live specimens that were collected, packaged, and shipped, as described in the protocol. Packages were shipped via standard UPS overnight shipping from OSUCCC-Lab and received at CerFlux-Lab within 24h of shipment. Hematoxylin and Eosin (H&E) staining verified the conserved structures of the nuclei and extracellular matrix, Kiel 67 (Ki-67) analysis revealed multiple areas of cellular proliferation in the tissue biospecimens, and Cleaved Caspase-3 staining showed minimal apoptotic sections. Moreover, received specimens continued to grow in culture thereby suggesting that tissue biospecimens not only remained viable after collection, handling, packaging, shipping, and receipt 24h later, they also maintained proliferation potential.

## ACKNOWLEDGEMENTS

This work was supported by grants from the National Science Foundation (TI-2321805) and the National Cancer Institute at the National Institutes of Health (1R43CA254493-01). We also thank our colleagues at The University of Virginia Comprehensive Cancer Center, The Holden Comprehensive Cancer Center, The O'Neal Comprehensive Cancer Center, and the Aga Khan University Nairobi Cancer Centre for their support and collaboration.

## CONFLICTS OF INTEREST

Dr. Budhwani is co-inventor of pending and issued patents pertaining to *in vitro*, *ex vivo*, and cancer supermodels.

## References

1. Fitzgibbons PL. Challenges in improving preanalytic specimen handling of routine cancer biospecimens. Arch Pathol Lab Med 2019;143(11):1300–1.
2. Compton CC, Robb JA, Anderson MW, et al. Preanalytics and precision pathology: Pathology practices to ensure molecular integrity of cancer patient biospecimens for precision medicine. Arch Pathol Lab Med 2019;143(11):1346–63.
3. Carithers LJ, Agarwal R, Guan P, et al. The biospecimen preanalytical variables program a multiassay comparison of effects of delay to fixation and fixation duration on nucleic acid quality. Arch Pathol Lab Med 2019;143(9):1106–18.
4. Abdollahi A, Saffar H, Saffar H. Types and frequency of errors during different phases of testing at a clinical medical laboratory of a teaching hospital in Tehran, Iran. N Am J Med Sci [Internet] 2014;6(5):224. Available from: http://www.najms.org/text.asp?2014/6/5/224/132941
5. Charania AA, Patel HK, Punjani Z, et al. Preanalytical protocols for improving access to live tissue diagnostics in remote and low resource settings. J Clin Oncol 2022;40.

## Figures

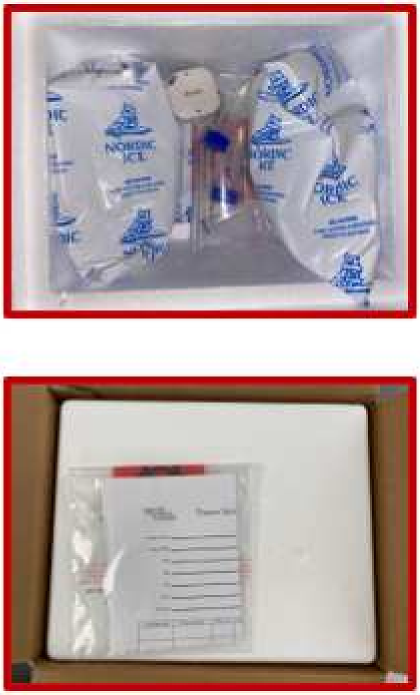

**Figure 1**

Unnumbered image in the Materials and equipment section.
